# The prevalence of herbal medicine among Sudanese adults: a cross-sectional study 2021

**DOI:** 10.1186/s12906-024-04584-1

**Published:** 2024-08-14

**Authors:** Raheeg Mohamed, Reham Mohamed, Rana Dafalla, Aya Ahmed, Abdulrahman Abdeldaim

**Affiliations:** Faculty of Medicine, University of Khartoum, ElQas Ave, Khartoum, Sudan

**Keywords:** Traditional medicine, Complementary medicine, Herbal medicine usage, Herbal remedies, Sudan

## Abstract

**Background:**

The use of herbal medicine has a long history in Sudan and is widely practiced among the general population. However, there is a lack of studies examining the prevalence, patterns, and predictors of herbal medicine usage in Sudan. Thus, this study was conducted to bridge this gap.

**Methods:**

This descriptive cross-sectional study was conducted between January and February 2021. It included Sudanese adults residing in Omdurman, Sudan, using systematic and simple random sampling methods. Data were collected using a structured, adapted questionnaire comprising: the socio-demographic characteristics and the knowledge of herbal medicine and its usage. Moreover, it investigated the commonly used herbal remedies and the participants’ sources of information and procurement of such products. Additionally, we examined the correlation between socio-demographic factors, cultural beliefs, and the use of herbal medicine. Data were analyzed using SPSS, and categorical data were presented as frequencies and percentages. Associations were assessed using chi-square, Fisher’s exact tests, and binary logistic regression (*p* < 0.05).

**Results:**

This study included 381 participants, of which 48.1% were females and 31.4% were aged 20-30. The majority of participants were aware of the practice of herbal medicine and the prevalence of its usage was 85.9%. Peppermint, acacia, hibiscus, ginger, and fenugreek were the most commonly used remedies. Chi-square and Fisher’s exact testing revealed that the participants’ gender and beliefs in the safety and effectiveness of herbal medicines were significantly associated with herbal medicine usage (*p* < 0.05). Binary logistic regression analysis showed that only the perception of herbal medicine’s safety was an independent predictor of its usage (p-value 0.038).

**Conclusion:**

This study reported a very high prevalence of herbal medicine usage, highlighting the acceptability of Sudanese adults towards herbal medicine usage. This prompts further studies to explore their safety, efficacy, and the possibility of their integration into mainstream healthcare practices and policies.

**Supplementary Information:**

The online version contains supplementary material available at 10.1186/s12906-024-04584-1.

## Background

Traditional medicine refers to the use of knowledge, skills, and practices that rely on the theories and beliefs of various cultures to maintain, prevent, diagnose, promote, and treat both physical and mental health [1, 2]. This age-old tradition finds its origin in countries with ancient civilizations such as China, India, Egypt, and South America [3]. Before the advent of modern medicine, traditional medicine held a prominent position as the primary healthcare system accessible to millions of individuals residing in African rural and urban areas [4]. In fact, it served as the sole healthcare option for a significant portion of the population. As per the 2019 WHO Global Report on Traditional and Complementary Medicine, different traditional medicine systems involve a range of practices such as acupuncture, herbal remedies, indigenous traditional medicine, homeopathy, traditional Chinese medicine, naturopathy, chiropractic, osteopathy, ayurvedic medicine, and Unani medicine [5]. Herbal medicine is an essential part of traditional medicine that relates to herbs, herbal materials, and herbal preparations, which are collectively referred to as herbal medicines. These products are made of different plant materials, like seeds, berries, roots, leaves, bark, or flowers, which contain active ingredients. Essentially, herbal medicines are natural products derived from plants that are used for medicinal purposes [6, 7].

Herbal medicines continue to be popular in developing countries because of the common belief in their safety, availability, affordability, and cultural acceptability. They are used as over-the-counter medications or recommended by a doctor or a pharmacist [8]. In Sudan herbal medicines are widely recognized for their role in preventing and treating various diseases. Numerous plant substances are used both as dietary components and for medicinal purposes. Sudan’s natural habitat and ecological characteristics provide an ideal environment for the growth of diverse herbs [9]. Traditional medicine remains the mainstay of healthcare for 90% of Sudanese population due to limited access to hospitals and conventional drugs especially in rural areas [10, 11].

It is important to note that while herbal medicines are generally considered safe, they can still carry risks and side effects. Reported adverse effects listed in previous studies range from relatively mild effects such as nausea, vomiting, diarrhea, constipation, and skin rash to more serious adverse effects such as elevated liver enzymes, hypokalemia, and reactions with cellular macromolecules, including DNA, causing cellular toxicity and/or genotoxicity [12, 13]. Understanding patterns, frequencies, and predictors of herbal medicine use can help healthcare providers identify individuals who may need extra guidance on using these remedies safely [14].

In light of this, there appears to be a need to conduct more cross-sectional research on the use of herbal medicine to gain a comprehensive understanding of the current prevalence, patterns, and predictors of herbal medicine usage within the Sudanese general population. This offers valuable insights into the characteristics and behaviors of Sudanese individuals who utilize herbal remedies. The findings can be used by healthcare providers and policymakers to identify emerging trends, associations, and potential risks associated with herbal medicine usage. This can lead to strict government regulations to promote the safe and responsible use of herbal remedies and raise public awareness regarding their use.

Previous studies on herbal medicine use were conducted across different counties including Turkey, Morrocco, Sierra Leone, Bangladesh, Nigeria, Palestine, Jordan, and Malaysia [6, 11, 13–19]. However, there is a lack of studies among the Sudanese population regarding the use of herbal medicine. Literature is scanty in estimating the frequency, patterns, and predictors of herbal medicine usage among the Sudanese general population. This study aims to examine the prevalence of herbal medicine usage among Sudanese adults in a residential district in Omdurman city, Khartoum, Sudan. The objectives include identifying the most commonly used Sudanese herbal remedies and their usage patterns for different medical conditions, evaluating the availability and accessibility of these remedies, and exploring the relationship between cultural beliefs and socio-demographic characteristics of Sudanese adults with the use of herbal medicines.

## Methods

### Study design

This is a descriptive, non-interventional, community-based, cross-sectional study aiming to investigate the prevalence and patterns of herbal medicine usage among Sudanese adults in a residential district in Omdurman city, Khartoum state. The study was conducted between January 2021 and February 2021.

### Study setting

The study was conducted in Omdurman City, which is located in the north-western region of Khartoum, Sudan. Omdurman has a population of approximately 2.5 million within an area of 614.9 km2.

### Study population

Households’ adults of the selected residential district in Omdurman city, Khartoum, during the period of the study were included in the study population.

### Inclusion and exclusion criteria

Both males and females who resided within the selected residential district during the period of the study were included in the study. Individuals who expressed a lack of interest and willingness to participate were excluded from the study.

### Sample size and sampling technique

The following formula was used to calculate the sample size [11]:

$$n_{0}=\frac{Z^{2}pq}{e^{2}}$$

Using an estimated prevalence of 50%, the sample size was estimated to be a minimum of 384. A total of 381 were enrolled in the study. The minimal sample size was not achieved.

The study used a two-stage sampling approach:

1. Systematic random sampling of houses:

- The authors had a list of all the residential houses in the target area of Omdurman City, which served as the sampling frame.
- They then used a systematic random sampling technique to select every 4th house from the list, ensuring a random selection of houses across the residential district.

2. Simple random sampling of individuals within the selected houses:

- Within each of the selected houses, the authors had a list of the individuals residing there.
- From this list of individuals, they used a simple random sampling technique to select the participants for the study.

### Data collection methods and tools

Data were collected using a structured, adapted, both open-and-close-ended, paper-and-pencil questionnaire over a period of one month (January 2021-February 2021). The questionnaire used in this study was adapted from previously validated instruments in the literature [11, 19]. To scrutinize the content validity of the adapted questionnaire, we had it reviewed by a panel of university professors with expertise in the research area. The professors provided feedback on the relevance, clarity, and comprehensiveness of the items. Based on their input, we refined the questionnaire items to improve the overall content validity of the instrument.

Before filling the questionnaire, subjects were asked to give an informed consent for participation by answering the question: I agree to participate in the study.

The questionnaire was structured into five sections:

- Section one contained questions considering the sociodemographic characteristics of the participants, including age, gender, marital status, level of education, occupation, and monthly family income.
- Section two contained questions regarding the knowledge of the participants about herbal medicines and their usage patterns.
- Section three explored the types of commonly used Sudanese herbal remedies, the forms in which they are used, and the medical conditions associated with their use.
- Section four contained questions related to sources of information about herbal medicines and sources for obtaining these products.
- Sections five focused on cultural beliefs and their association with the use of herbal medicine.

The questionnaire used in this study is available as Supplementary File 1.

### Data management and analysis

Questionnaires were refined and managed carefully before data entry. Data were tabulated, entered into a Microsoft Excel database, and analyzed using the statistical package for social sciences program version 21 (SPSS). Categorical data were expressed as frequencies and percentages. Numerical data were presented as the mean  ±  standard deviation. The association between the use of herbal medicine and the characteristics of the participants was tested using the chi-square test, Fisher’s exact test and binary logistic regression model with a statistically significant p-value of < 0.05.

## Results

### Sociodemographic characteristics

One-third of the participants (33.4%) were between 20 and 30 years old. More than one-third (46.3%) were married and had an income less than 10,000 SDG per month (42.8%). More than half of the subjects had university education (55.4%) (Table 1).

**Table 1:** Descriptive Sociodemographic characteristics of the participants

Variable		*n*	%
Age group (In years)	Less than 20	23	6.1
	20–30	118	31.4
	31–40	52	13.8
	41–50	67	17.8
	More than 50	116	30.9
Gender	Male	196	51.9
	Female	182	48.1
Marital status	Unmarried	161	42.6
	Married	175	46.3
	Separated	7	1.9
	Divorced	11	2.9
	Widowed	24	6.3
Education	Unformal	12	3.2
	Primary	14	3.7
	Secondary	53	14.1
	University	209	55.4
	Above university	89	23.6
Occupation	Student	80	21.2
	Public Servant	55	14.6
	Private Employer	56	14.9
	Free Lancer	62	16.4
	Worker	8	2.1
	Housewife	82	21.8
	Others	34	9.0
Monthly Income (SDG)	Less than 10000	137	42.8
	10000–20000	84	26.3
	More than 20000	99	30.9

We observed a statistically significant association between the participants’ gender and their usage of herbal medicines (Table 4).

**Table 2:** Knowledge and patterns of use of herbal medicines among the participants

		*n*	%
Are you aware of using natural health products?	Yes	347	91.6
	No	32	8.4
Do you know what herbal medicine is?	Yes	337	89.4
	No	40	10.6
Do you use herbal medicines?	Yes	323	85.9
	No	53	14.1
How often have you used herbal medicines over the past year?	Daily	40	12.1
	Sometimes	97	29.4
	When sick	187	56.7
	Never	6	1.8
In which form do you use herbal medicines?	Herbal Tea	263	69
	Herbal Juice	61	16
	Herbal Extract	48	12.6
	Herbal Oil	137	36
	Herbal Powder	119	31.2
	Herbal Supplements	55	14.4
	Herbal Fumes	164	43
	Others	17	4.5
For which purposes do you use herbal medicines?	Health promotion	114	29.9
	Prevention of diseases	156	40.9
	Treatment of diseases	261	68.5
	I don’t use herbal medicines	5	1.3
	Others	9	2.4

### Knowledge and patterns of use of herbal medicines

The majority of the participants (91.6%) were aware of using natural health products. Most of them knew “what herbal medicine is” (89.4%) and reported using herbal medicines (85.9%). More than half of the participants (56.7%) declared using herbal medicines “when sick.” The most common way to use herbal remedies is by preparing them in the form of tea (69%) (Table 2).

**Table 3:** Types and frequencies of the most commonly used Sudanese herbal remedies

Type of Herbal Remedy	%
Peppermint (Mentha piperita L.)	73.8
Gum Arabic Tree (Acacia nilotica)	71.4
Hibiscus (Hibiscus sabdariffa L.)	70.9
Fenugreek (Trigonella foenum-graecum L.)	66.9
Ginger (Zingiberaceae Zingiber officinale)	69.8
Baobabs (Adansonia)	66.4
Black cumin (Nigella sativa)	58.5
Cinnamon (Cinnamomum verum)	57.5
Clove (Syzygium aromaticum)	56.4
Tamarind (Tamarindus indica)	54.9
Solenostemma (Solenostemma argel)	50.4
Doum Palm (Hyphaene thebaica)	45.1
Guddaim Fruits (Grewia Tenax)	41.5
Green Tea	41.2
Lemongrass (Cymbopogon citratus)	40.2
Henna (Lawsonia inermis)	35.7
Acacia	32.5
Sidr (Ziziphus spina-christi)	31
Aloe Vera	29.4
Neem (Azadirachta indica)	19.4

### Types of herbal remedies used

Table 3 illustrates the frequencies of the most commonly used Sudanese herbal remedies. The top five herbal remedies used were peppermint (73.8%), acacia nilotica (71.4%), hibiscus (79.9%), ginger (69.8%), and fenugreek (66.9%).

**Table 4:** Sociodemographic characteristics of the participants and their association with herbal medicine usage

Variable		Herbal Medicine Use Yes No		*P-value*
Age groups	Less than 20	16	5	0.412
	20–30	97	20	
	31–40	45	7	
	41–50	60	7	
	More than 50	101	13	
Gender	Male	156	37	**0.004**
	Female	165	16	
Marital status	Married	153	27	0.223
	Separated	4	21	
	Divorced	10	2	
	Widowed	22	1	
	Unmarried	132	1	
Education	Unformal	8	3	0.445
	Primary level	13	1	
	Secondary level	47	6	
	University level	179	27	
Occupation	Student	61	17	0.229
	Government employer	47	8	
	Private sector employer	48	7	
	Self-employed professional	52	10	
	Worker	8	0	
	Unemployed	73	18	
Monthly Income (SDG)	Less than 10000	115	20	0.685
	10000–20000	75	9	
	More than 20000	85	13	

*Abbreviation* SDG Sudanese pound

### Common medical conditions for which herbal remedies are widely used

**Fig. 1:**
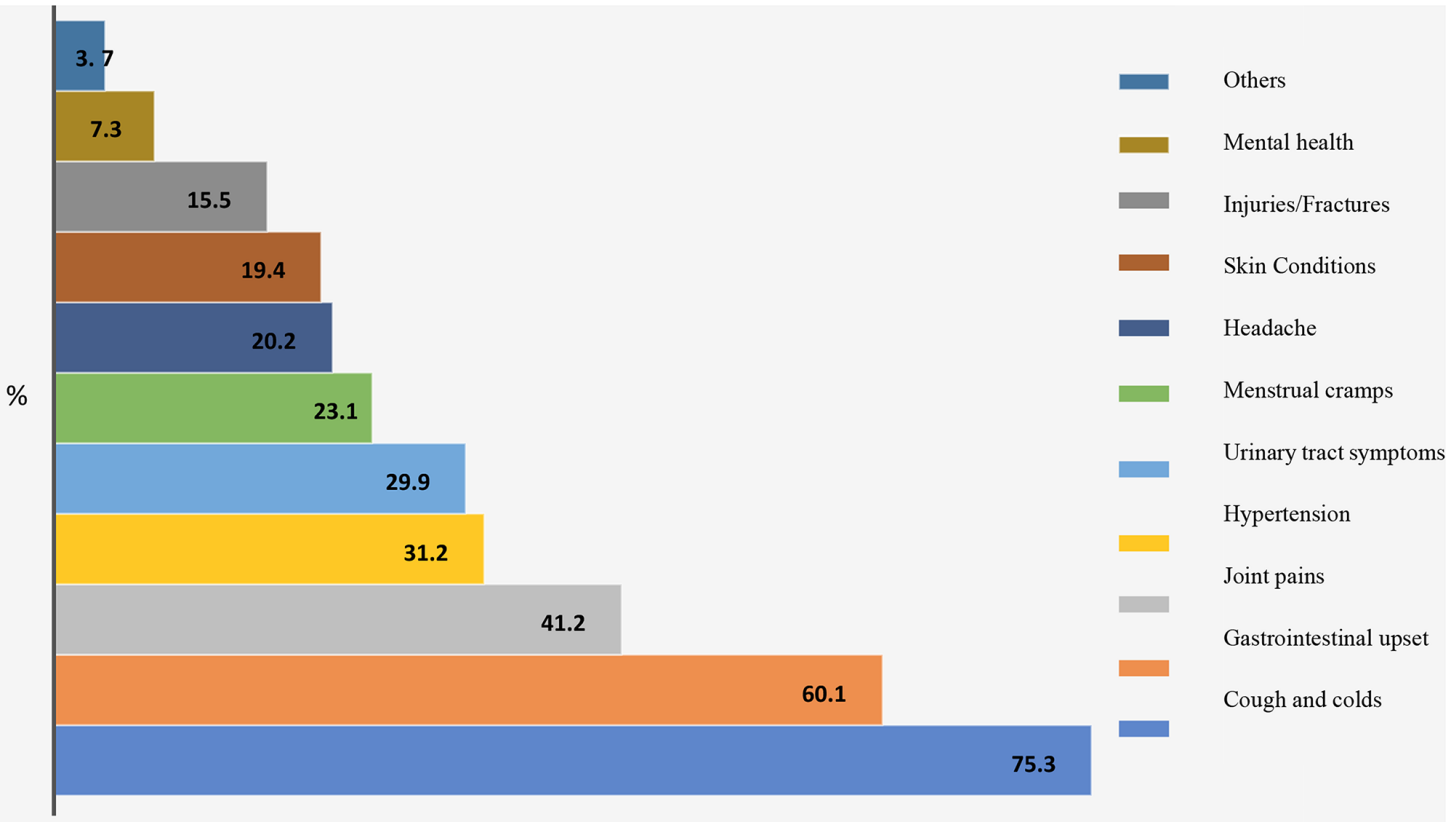
Common medical conditions for which herbal remedies are widely used

As Fig. 1 shows, herbal remedies are used for treating various medical conditions. The top three were coughs and colds (75.3%), gastrointestinal disturbances (60.1%), and joint pains (41.2%).

### Sources of information about how to obtain herbal remedies

**Fig. 2:**
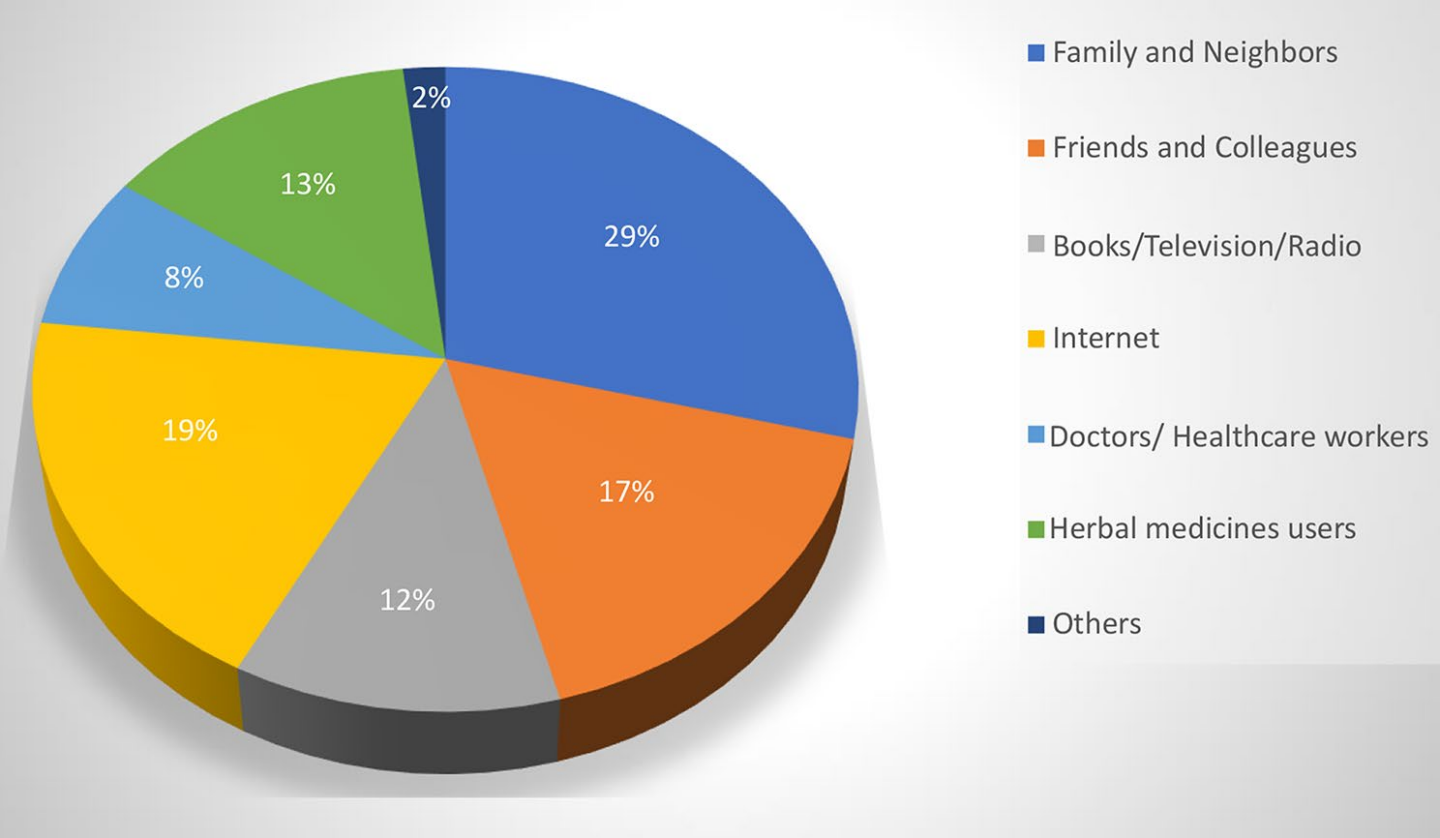
Sources of information about how to obtain herbal remedies

Figure 2 shows that the main sources of information about how to obtain herbal medicines are families and neighbors (29%), the internet (19%), followed by friends and colleagues (17%).

### Sources for obtaining herbal remedies among the participants

**Fig. 3:**
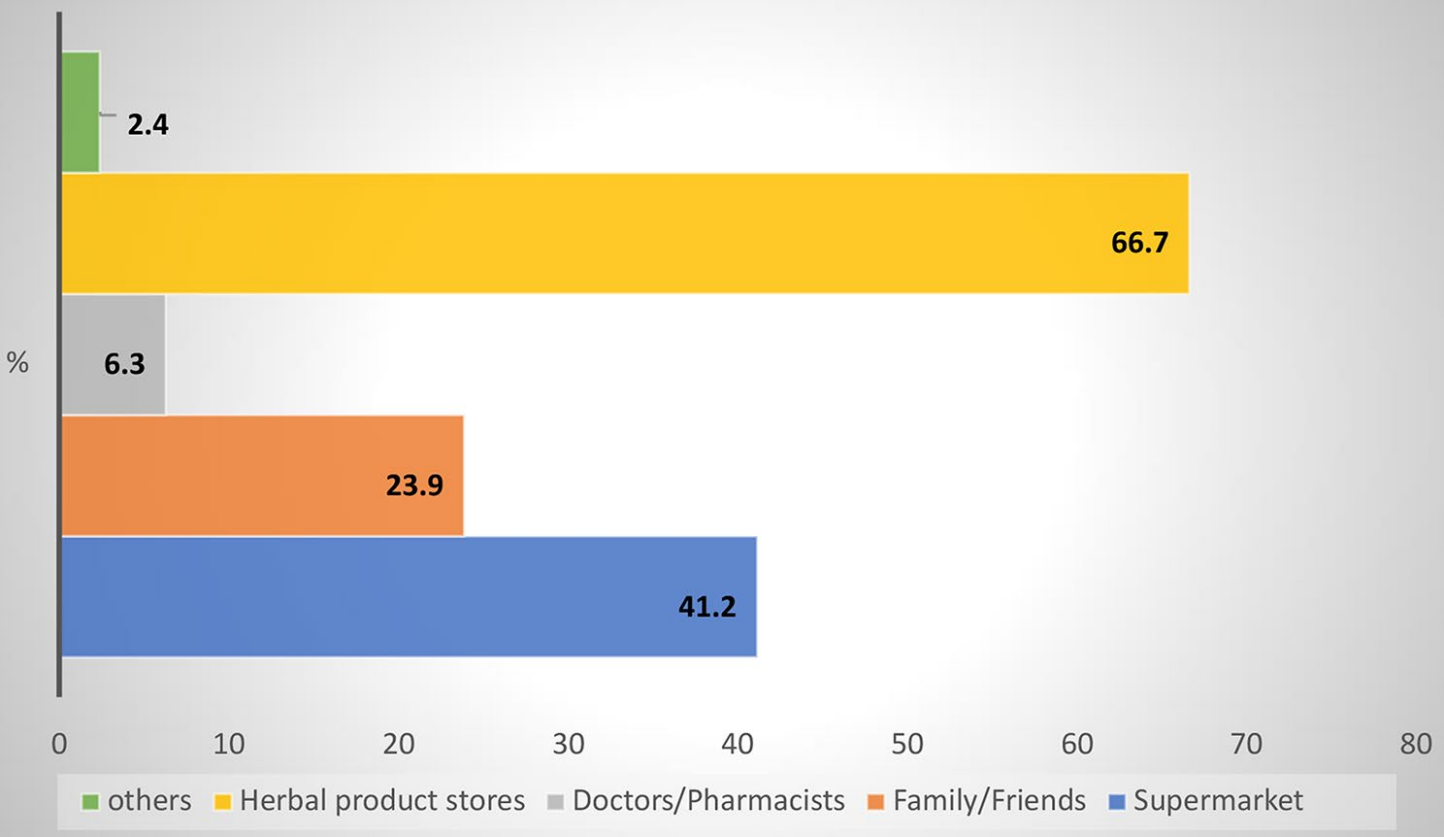
Sources for obtaining herbal remedies among the participants

The two most prevalent sources to obtain herbal remedies, as Fig. 3 displays, were herbal product stores and supermarkets.

### Beliefs about herbal medicines among the participants

Most of the participants believed that herbal medicines have fewer side effects, are safer, and are more effective in treating various diseases (Table 5).

**Table 5:** Beliefs about herbal medicines among the participants and their association with herbal medicine usage

		Herbal medicine use		*P-value*
		Yes	No	
Herbal medicines have lesser side effects	Yes	269	4	0.571
	No	49	1	
Herbal medicines are safer	Yes	200	2	**0.006**
	No	27	3	
Herbal medicines are cheaper	Yes	133	2	1.00
	No	138	3	
Herbal medicines are more effective in treating diseases	Yes	301	3	**0.027**

We found a statistically significant association between the beliefs of the participants about the safety of herbal medicines and their usage of herbal medicine (Table 5).

We also observed a statistically significant association between the beliefs of the participants about the effectiveness of herbal medicines and their usage of herbal medicine (Table 5).

### Binary logistic regression analysis for factors associated with herbal medicine usage

Factors demonstrating significant association with herbal medicine usage in the univariate analysis were included in a binary logistic regression model; only perception of herbal medicine’s safety was an independent predictor of their use (p-value 0.038, odds ratio 9.65 (Table 6).

**Table 6:** Binary logistic regression for factors associated with herbal medicine usage

	Odds ratio	*P-value*
Gender	1.172	0.869
Herbal medicines are safer	9.653	0.038
Herbal medicines are more effective in treating diseases	3.438	0.278

## Discussion

Several studies have documented the use of herbal medicine in specific health subpopulations and various locations and settings [15, 16, 18, 20]. The use of herbal medicine by the general population has been reported in Nigeria, Jordan, and Malaysia [6, 11, 13]. However, there is limited information available on the prevalence of herbal medicine usage in the general population within Sudan.

This study examined the prevalence of herbal medicine usage among Sudanese adults in a residential district in Omdurman city, Khartoum. A high prevalence of 85.9% was observed in our study. This is close to a similar rate observed in another study from Jordan [13], but is considered a higher rate compared to findings presented in other studies from Nigeria, and Malaysia [6, 13]. This can be attributed to the cultural and traditional acceptability of herbal remedies and the availability of diverse herbal resources. Sudan’s geographical location and climate provide a rich environment for a diverse range of medicinal plants and herbs, making them easily accessible to the population.

The study identified the most commonly used Sudanese herbal remedies, including peppermint, acacia nilotica, hibiscus, ginger, and fenugreek. This highlights that these herbal remedies hold cultural significance and are perceived as effective in managing various health conditions within the Sudanese population. Further research and exploration of these herbal remedies can provide valuable insights into their safety, efficacy, and potential integration into mainstream healthcare practices.

A significant proportion of the participants (56.7%) reported using herbal medicines, primarily when sick. The most common method of using herbal remedies was preparing them in the form of tea, which aligns with traditional practices in the region. Herbal remedies were found to be commonly used for treating coughs and colds, gastrointestinal disturbances, and joint pains, in contrast to a study in Nigeria where they were frequently used for treating malaria and reducing blood sugar levels. Another study done in Morocco showed that they were more frequently used for gastrointestinal disorders, which aligns with our findings [15]. This suggests that herbal medicines are perceived as effective remedies for these medical conditions among the participants. Further research is needed to explore the specific mechanisms and potential benefits of these herbal remedies for the mentioned conditions. Understanding the active compounds, pharmacological properties, and potential interactions with conventional treatments can provide valuable insights into their effectiveness and safety. Clinical trials and rigorous scientific studies are necessary to evaluate their efficacy and establish evidence-based guidelines for the use of these herbal remedies in the management of these medical conditions.

The main sources for obtaining herbal remedies were herbal product stores and supermarkets. This finding highlights the accessibility and availability of herbal medicines through commercial channels. Additional studies are need to ensure the quality and safety of herbal products obtained from these sources. The main sources of information on how to obtain herbal remedies were families, neighbors, and the internet which is consistent with the findings of some previous studies [12, 15]. This highlights the influence of social networks and the growing role of online platforms in shaping the use of herbal medicines in the Sudanese population.

This study revealed a statistically significant association between the participants’ gender and the use of herbal medicine, with a p-value of 0.004, indicating that women were more likely to use these remedies than men, which is consistent with the findings of various previous studies [14, 21–23]. This could be due to multiple factors. One possible explanation is that women often play a central role in family healthcare and are more likely to seek alternative remedies for themselves and their families. Women may also have specific health concerns or conditions for which they find herbal remedies more suitable or effective. This discrepancy may also be due to the differences in how females and males perceive and define herbal medicines, which can contribute to variations in how these remedies are recognized and valued. These gender-based variations can help explain the differences observed in the use of herbal medicine. Attitudes towards overall health can also contribute to explain this gender difference [14]. However, further research is needed to explore the specific reasons behind the gender disparity in herbal medicine usage.

The participants’ beliefs about herbal medicines were found to be significantly associated with their actual usage. The belief that herbal medicines are safer and more effective in treating various diseases appeared to influence the decision to use herbal remedies, with p-values of 0.006 and 0.027, respectively. These beliefs reflect the perception of herbal medicines as a natural and holistic approach to healthcare, aligning with traditional Sudanese healing practices. It could also be explained by the historical context of herbal medicines in Islamic culture, which established a strong acceptance of these products among users, enhancing their credibility and popularity [12]. The perception of herbal medicine’s safety was the only independent predictor of their use (p-value = 0.038, OR = 9.65). This finding suggests that the participants’ beliefs about the safety of herbal remedies played a crucial role in determining whether they chose to use these products, even after accounting for other potential factors. This highlights the importance of addressing concerns about the safety of herbal products and promoting their credibility as a means of encouraging their uptake among the study population. Strategies to enhance the perceived safety of herbal medicines, such as increased regulation, quality control, and public education, may be particularly effective in driving their usage in the Sudanese context, where traditional healing practices remain deeply embedded in the cultural fabric.

This study has several limitations that warrant acknowledgement. While the study aimed to have a robust sample size, the minimal sample size required for the analysis was ultimately not achieved. The use of a general prevalence of 50% for herbal medicine usage among Sudanese adults, rather than relying on prevalence data from other studies, may have also introduced some uncertainty or bias in the estimation of the true prevalence within the target population. To address these limitations, future research would benefit from conducting a comprehensive systematic review or meta-analysis of studies on herbal medicine usage in the Sudanese context. This could provide more reliable and context-specific prevalence data to inform the design and analysis of subsequent studies with an adequately powered sample size. The study was conducted in only one residential district in Omdurman city, Khartoum. Therefore, the findings may not be representative of the entire population or other residential districts and rural areas. Generalizing the results to a broader population or different geographic localities should be done with caution. In order to ensure that the findings are applicable to a wider population, future research should cover multiple districts and diverse areas.

The study was done during the COVID-19 pandemic period; therefore, due to health restrictions, a paper-and-pencil questionnaire was adopted instead of one-to-one direct interviews with the participants. Using a paper-and-pencil questionnaire as the data collection method in this study has two limitations: the potential for missing data due to skipped or omitted questions and the possibility of inaccurate responses influenced by social desirability bias, memory recall limitations, or question misunderstanding. These limitations may have affected the completeness, accuracy, and generalizability of the data. To mitigate these limitations, future research should explore strategies to minimize missing data, consider alternative data collection methods, and enhance participants’ understanding of the questionnaire. In addition, although the content validity of the adapted questionnaire was established through the review and feedback from university professors, the reliability and construct validity of the instrument were not formally tested in this study. Future research should consider evaluating the psychometric properties of the adapted questionnaire to further strengthen the validity and reliability of the measurement tool.

This study focused on the use of herbal medicines for a limited number of medical conditions, such as coughs and colds, gastrointestinal disturbances, and joint pains. Other medical conditions were not evaluated, which may limit the generalizability of the findings to a broader range of health conditions. It is possible that herbal medicines may be used differently or have varying efficacy for other medical conditions not assessed in this study. Therefore, further research is needed to explore the use of herbal medicines for a wider spectrum of health conditions to provide a more comprehensive understanding of their potential benefits and limitations.

Another limitation of the study is the lack of a clear link between the use of specific herbal remedies for treatment of specific medical conditions. This absence of specificity hinders accurate conclusions regarding the effectiveness and appropriateness of herbal remedies for specific conditions. Consequently, the study’s findings may have limited practical implications for healthcare providers and individuals seeking evidence-based guidance on herbal medicine usage. Future research should aim to establish clearer connections between specific herbal remedies and their corresponding medical indications to enhance understanding of their therapeutic potential. In addition to this, the study did not evaluate the effect of concurrent use of conventional medicine with herbal medicines, which may have influenced the participants’ perceptions of effectiveness, safety, outcomes, and satisfaction levels. Another limitation is that the data collected in the study provide a snapshot of the participants’ experiences with herbal medicines at a specific point in time. It is unable to capture any changes or developments in their experiences over time.

## Conclusion

The study revealed a high prevalence rate of herbal medicine use among Sudanese adults (85.9%), which reflects the cultural acceptability and availability of diverse herbal resources in Sudan. The most commonly used herbal remedies are peppermint (73.8%), acacia nilotica (71.4%), hibiscus (79.9%), ginger (69.8%), and fenugreek (66.9%). The findings also highlight that herbal medicines are commonly used for treating coughs and colds (75.3%), gastrointestinal disturbances (60.1%), and joint pains (41.2%). The preferred method of using herbal remedies is preparing them as tea (69%), which aligns with traditional practices in the region. The study also revealed that women were more likely to use herbal remedies than men (p-value 0.004). The beliefs about herbal medicines, particularly their perceived safety (p-value 0.006) and effectiveness in treating various diseases (p-value 0.027), may influence the decision on their usage. The perception of herbal medicine’s safety was the only independent predictor of their use (*p* = 0.038, OR = 9.65). Further research is needed to explore the safety, efficacy, and potential integration of these remedies into mainstream healthcare practices. It is essential to ensure the quality of herbal products obtained from commercial sources while also considering the influence of social networks and online platforms in shaping herbal medicine usage. By gaining a deeper understanding of these aspects, evidence-based guidelines can be established to promote the safe and effective use of these remedies in managing various medical conditions.

### Electronic supplementary material

Below is the link to the electronic supplementary material.

Supplementary Material 1

## Data Availability

The corresponding author can provide the datasets used and analyzed in this study upon reasonable request.

## References

[1] Fong HHS. Integration of herbal medicine into modern medical practices: issues and prospects. Integrative Cancer Therapies. Volume 1. SAGE Publications Inc.; 2002. pp. 287–93. 10.1177/153473540200100313 14667286

[2] Sharma A, Shanker C, Tyagi LK, Singh M, Rao CV. Herbal Medicine for Market potential in India: an overview. Acad J Plant Sci. 2008;1(2):26–36.

[3] Abdullahi AA. Trends and challenges of traditional medicine in Africa. Afr J Tradit Complement Altern Med. 2011;8(5 SUPPL):115–23. 22754064 10.4314/ajtcam.v8i5S.5 PMC3252714

[4] World Health Organization. WHO global report on traditional and complementary medicine 2019. 226 p.

[5] Traditional medicine Report by the Secretariat [Internet]. http://www.who.int/.

[6] Oreagba IA, Adeola Oshikoya K, Amachree M. Herbal medicine use among urban residents in Lagos, Nigeria [Internet]. 2011. http://www.biomedcentral.com/1472-6882/11/117. 10.1186/1472-6882-11-117 PMC3252251 22117933

[7] Rafieian-Kopaei M, Sewell RDE, R T I C L E I N F O [Internet]. The history and ups and downs of herbal medicines usage A. Vol. 3, Journal of HerbMed Pharmacology Journal homepage: J HerbMed Pharmacol. 2014. http://www.herbmedpharmacol.com.

[8] Hala AA, Musa BB, Eltayeb Elhag Ali Co-supervisor, Abdel Rahman Ali S. H. Quality of medicinal plants traditionally used in Sudan as affected by ionizing radiation treatments. M.Sc. (Agric.). 1994.

[9] Elegami AA, El-Nima EI, El Tohami MS, Muddathir AK. Antimicrobial activity of some species of the family combretaceae. Phytother Res. 2002;16(6):555–61. 10.1002/ptr.995 12237814

[10] Koko WS, Galal M, Khalid HS. Fasciolicidal efficacy of Albizia anthelmintica and Balanites aegyptiaca compared with albendazole [Internet]. Vol. 71, Journal of Ethnopharmacology. 2000. www.elsevier.com/locate/jethpharm. 10.1016/s0378-8741(00)00172-0 10904170

[11] El-Dahiyat F, Rashrash M, Abuhamdah S, Abu Farha R, Babar ZUD. Herbal medicines: a cross-sectional study to evaluate the prevalence and predictors of use among Jordanian adults. J Pharm Policy Pract. 2020;13(1). 10.1186/s40545-019-0200-3 PMC6971905 31988754

[12] Ekor M. The growing use of herbal medicines: issues relating to adverse reactions and challenges in monitoring safety. 4 JAN, Front Neurol. 2014. 10.3389/fphar.2013.00177 PMC3887317 24454289

[13] Aziz Z, Tey NP. Herbal medicines: prevalence and predictors of use among Malaysian adults. Complement Ther Med. 2009;17(1):44–50. 19114228 10.1016/j.ctim.2008.04.008

[14] Tulunay M, Aypak C, Yikilkan H, Gorpelioglu S. Herbal medicine use among Turkish patients with chronic diseases. J Intercult Ethnopharmacol. 2015;4(3):217. 26401410 10.5455/jice.20150623090040 PMC4579486

[15] Touiti N, Houssaini TS, Iken I, Benslimane A, Achour S. Prevalence of herbal medicine use among patients with kidney disease: a cross-sectional study from Morocco. Nephrologie et Therapeutique. 2020;16(1):43–9. 31383617 10.1016/j.nephro.2019.01.007

[16] James PB, Kamara H, Bah AJ, Steel A, Wardle J. Herbal medicine use among hypertensive patients attending public and private health facilities in Freetown Sierra Leone. Complement Ther Clin Pract. 2018;31:7–15. 29705483 10.1016/j.ctcp.2018.01.001

[17] Ahmed M, Hwang JH, Hasan MA, Han D. Herbal medicine use by pregnant women in Bangladesh: a cross-sectional study. BMC Complement Altern Med. 2018;18(1). 10.1186/s12906-018-2399-y PMC6293557 30545348

[18] Aina O, Gautam L, Simkhada P, Hall S. Prevalence, determinants and knowledge about herbal medicine and non-hospital utilisation in southwest Nigeria: a cross-sectional study. BMJ Open. 2020;10(9). 10.1136/bmjopen-2020-040769 PMC7485235 32912997

[19] Quzmar Y, Istiatieh Z, Nabulsi H, Zyoud SH, Al-Jabi SW. The use of complementary and alternative medicine during pregnancy: a cross-sectional study from Palestine. BMC Complement Med Ther. 2021;21(1). 10.1186/s12906-021-03280-8 PMC8017862 33794888

[20] General. Guidelines for Methodologies on Research and Evaluation of Traditional Medicine. 2000.

[21] Gunther S, Patterson RE, Kristal AR, Stratton KL, White E. Demographic and health-related correlates of herbal and specialty supplement use. J Am Diet Assoc. 2004;104(1):27–34. 14702580 10.1016/j.jada.2003.10.009

[22] Lim MK, Sadarangani P, Chan HL, Heng JY. Complementary and alternative medicine use in multiracial Singapore. Complement Ther Med. 2005;13(1):16–24. 15907674 10.1016/j.ctim.2004.11.002

[23] Dole EJ, Rhyne RL, Zeilmann CA, Skipper BJ, McCabe ML, Dog TL. The influence of ethnicity on use of herbal remedies in elderly hispanics and non-hispanic whites. J Am Pharm Assoc (Wash). 2000;40(3):359–65. 10853536 10.1016/S1086-5802(16)31083-X

