# Supplementary material for: The prevalence of herbal medicine among Sudanese adults: a cross-sectional study 2021
Source: BMC Complement Med Ther. 2024 Aug 14;24:308. doi: 10.1186/s12906-024-04584-1 (PMC11325702; doi:10.1186/s12906-024-04584-1)
Supplement: Supplementary file 1 — Supplementary Material 1 [file 12906_2024_4584_MOESM1_ESM.pdf]

University of Khartoum

Faculty of Medicine

Department of Community Medicine

The use of herbal medicine among Sudanese adults: a cross-sectional study 2021.

Attention:

The questions in this questionnaire are for research purposes only, and the information you provide will be used solely for the purposes of this study. All responses will be kept strictly confidential, and your personal information will not be shared or used for any other purposes. Your participation is completely voluntary, and you have the full right to agree or decline to fill out the questionnaire below.

1- I agree ☐

2- I don't agree ☐

Form No. ☐

Age: ☐

Gender:

1- Male ☐

2- Female ☐

Marital Status:

1- Married ☐

2- Un-Married ☐

3- Separated ☐

4- Divorced ☐

5- Widowed ☐

Education:

1- Unformal ☐

2- Primary ☐

3- Secondary ☐

4- University ☐

5- Above university ☐

Occupation:

1- Student ☐

2- Public servant ☐

3- Private employer ☐

4- Free lancer ☐

5- Worker ☐

6- Housewife ☐

7- Others, specify: ☐

………
………

Monthly income in Sudanese pound:

1- Less than 10,000 ☐

2- 10,000 – 20,000 ☐

3- More than 20,000 ☐

1- Are you aware of using natural health products?

1- Yes ☐
2- No ☐

2- Do you know what Herbal Medicine is?

1- Yes ☐
2- No ☐

3- Do you use herbal medicines?

1- Yes ☐
2- No ☐

If your answer to the above question is yes, you can continue answering the following questions:

4- How often have you used herbal medicines over the past year?

1- Daily ☐
2- Sometimes ☐
3- When sick ☐
4- Never ☐

5- In which form do you use herbal medicines? (Please select all that apply).

1- Herbal tea or solution ☐
2- Herbal juice ☐
3- Herbal extract ☐
4- Herbal oil or cream ☐
5- Herbal powder ☐
6- Herbal supplement ☐
7- Herbal fumes ☐
8- Others, specify:
…………………………………………………………………………………………………
…………………………………………………………………………………………………

6- For which purposes do you use herbal medicines? (Please select all that apply).

1- Health Promotion ☐
2- Prevention of Diseases ☐
3- Treatment of Diseases ☐
4- Others, Specify:
…………………………………………………………………………………………………
…………………………………………………………………………………………………

7- The list below includes some of the most commonly used Sudanese herbal remedies. Please indicate which of these herbal products you have personally used or incorporated into your healthcare practices (Please select all that apply).

1- Acacia ☐

2- Lemongrass ☐

3- Solenostemma ☐

4- Gum Arabic Tree (Acacia nilotica) ☐

5- Aloe vera ☐

6- Baobabs ☐

7- Guddaim Fruits ☐

8- Doum Palm ☐

9- Hibiscus ☐

10-Fenugreek ☐

11-Tamarind ☐

12-Mint ☐

13-Ginger ☐

14-Cinnamon ☐

15-Sidr ☐

16-Henna ☐

17-Cloves ☐

18-Black cumin ☐

19-Green tea ☐

20-Neem ☐

21- Others, specify:

………………………………………………………………………………………………………
………………………………………………………………………………………………………

8- For which health conditions or symptoms do you use herbal remedies? (Please select all that apply)

1-Cough/Colds ☐

2-Hypertension ☐

3-Headache/Migraine ☐

4-Joints pain or inflammation ☐

5-Gastrointestinal disturbances ☐

6-Menstrual cramps and uterine conditions ☐

7-Injuries/Fractures ☐

8-Urinary tract disturbances ☐

9-Mental Health ☐

10-Skin Conditions ☐

11-Others, specify:

……
……

9- How do you obtain or access information about herbal remedies and their availability? (Please select all that apply)

1-Family/Neighbors ☐

2-Friends/Colleagues ☐

3-Books/Television/Radio ☐

4-Internet ☐

5-Doctors and healthcare workers ☐

6-Herbal medicines users ☐

7-Others, specify:

……
……

10- Where do you obtain or purchase the herbal medicines and remedies that you use? (Please select all that apply)

1-Supermarket ☐

2-Family/Friends ☐

3-Doctors/Pharmacist ☐

4-Herbal product stores ☐

5-Others, specify:

……
……

11-Herbal medicines are more effective in treating diseases, do you agree?

1-Yes ☐

2-No ☐

12- Herbal medicines are cheaper, do you agree?

1-Yes ☐

2-No ☐

13- Herbal medicines are safer, do you agree?

1-Yes ☐

2-No ☐

14- Herbal medicines have lesser side effects, do you agree?

1-Yes ☐

2-No ☐
